# Assessing targeted invitation and response modes to improve survey participation in a diverse New York City panel: Healthy NYC

**DOI:** 10.1371/journal.pone.0280911

**Published:** 2023-01-26

**Authors:** Shabitri Dasgupta, Sharon E. Perlman, Amber Levanon Seligson, Sarah E. Dumas, Kacie Seil, Tashema Bholanath

**Affiliations:** Division of Epidemiology, New York City Department of Health and Mental Hygiene, Long Island City, NY, United States of America; University of Bedfordshire, UNITED KINGDOM

## Abstract

**Background:**

Healthy NYC is an innovative survey panel created by the New York City (NYC) Department of Health and Mental Hygiene (DOHMH) that offers a cost-effective mechanism for collecting priority and timely health information. Between November 2020 and June 2021, invitations for six different surveys were sent to Healthy NYC panelists by postal mail, email, and text messages. Panelists had the option to complete surveys online or via paper survey.

**Methods:**

We analyzed whether panelists varied by sociodemographic characteristics based on the contact mode they provided and the type of invitation that led to their response using logistic regression models. Poisson regression models were used to determine whether the number of invitations received before participating in a survey was associated with sociodemographic characteristics.

**Results:**

Younger age and higher education were positively associated with providing an email or text contact. Furthermore, age, race, and income were significant predictors for invitation modes that led to a survey response. Black panelists had 72% greater odds (OR 1.72 95% CI: 1.11–2.68) of responding to a mail invite and 33% lesser odds (OR 0.67, 95% CI: 0.54–0.83) of responding to an email invite compared with White panelists. Additionally, in five of the six surveys, more than half of the respondents completed surveys after two invites. Email invitations garnered the highest participation rates.

**Conclusions:**

We recommend using targeted invitation modes as an additional strategy to improve participation in panels. For lower-income panelists who do not provide an email address, it may be reasonable to offer additional response options that do not require internet access. Our study’s findings provide insight into how panels can tailor outreach to panelists, especially among underrepresented groups, in the most economical and efficient ways.

## Introduction

Healthy NYC is an innovative local survey panel created by the New York City (NYC) Department of Health and Mental Hygiene’s (DOHMH) Division of Epidemiology in the spring of 2020. Healthy NYC provides a cost-effective mechanism for providing priority and timely health information [1]. Since the panel’s inception, Healthy NYC surveys have primarily focused on experiences during and consequences of the COVID-19 pandemic. They have provided information on the prevalence of COVID-like illness [2]; opinions about treatment, testing, and vaccination; awareness of governmental recommendations; mental health [3] and financial consequences of the pandemic; and associated racial inequities [4].

While Healthy NYC is a probabilistically constructed panel, encouraging participation in Healthy NYC surveys in ways that maintain representativeness remains a key concern. This is a particular concern in health-related surveys because non-respondents reportedly have a higher prevalence of risky health behaviors and poor self-rated health [5–10]. Previous research has found that the type of contact information provided [11, 12] and the likelihood of responding to surveys are associated with sociodemographic characteristics [13–16]. This suggests that inviting panelists using multiple forms of contact information and offering multiple response modes may increase a survey’s representativeness. In addition, the number of invitations panelists receive for each survey may motivate them differently and fine-tuning the contact frequency may help to encourage participation. There is currently no consensus in the literature on the optimal number of email invite reminders; however, the first few invites generally garnered the most responses [17–21].

As of April 2021, Healthy NYC was composed of 9,583 NYC residents 18 years and older who had been enrolled through two recruitment efforts in June and September 2020. This paper investigates three questions. First, do panelists vary by their sociodemographic characteristics based on the types of contact information they provide? Second, are panelists’ sociodemographic characteristics associated with their responses to invitations received by mail, text, and email? Third, do panelists vary by sociodemographic characteristics and the number of invitations received before participating in a survey? Understanding these components can help us produce targeted invitation systems that result in higher participation rates, more representative surveys, and more satisfied panelists.

## Methods

### Data collection and sampling

Non-institutionalized NYC residents ages 18 and older were probabilistically selected to be invited to join Healthy NYC, primarily from address-based samples, supplemented by those who had previously completed DOHMH probability-based surveys between 2019 and 2020 and agreed to be contacted for future research [1]. In the address-based samples, households were randomly sampled from a list of all residential addresses in NYC. In the samples from respondents to other DOHMH surveys, those respondents had been selected for the initial DOHMH surveys through Random Digit Dialing (RDD) of landline and cell phone numbers, or though random selection of municipal administrative records. To obtain intra-household randomization, the next birthday method was used. This meant that the person opening the invitation was asked to have the adult member of the household who would have the next birthday complete the panel registration survey.

**Fig 1:**
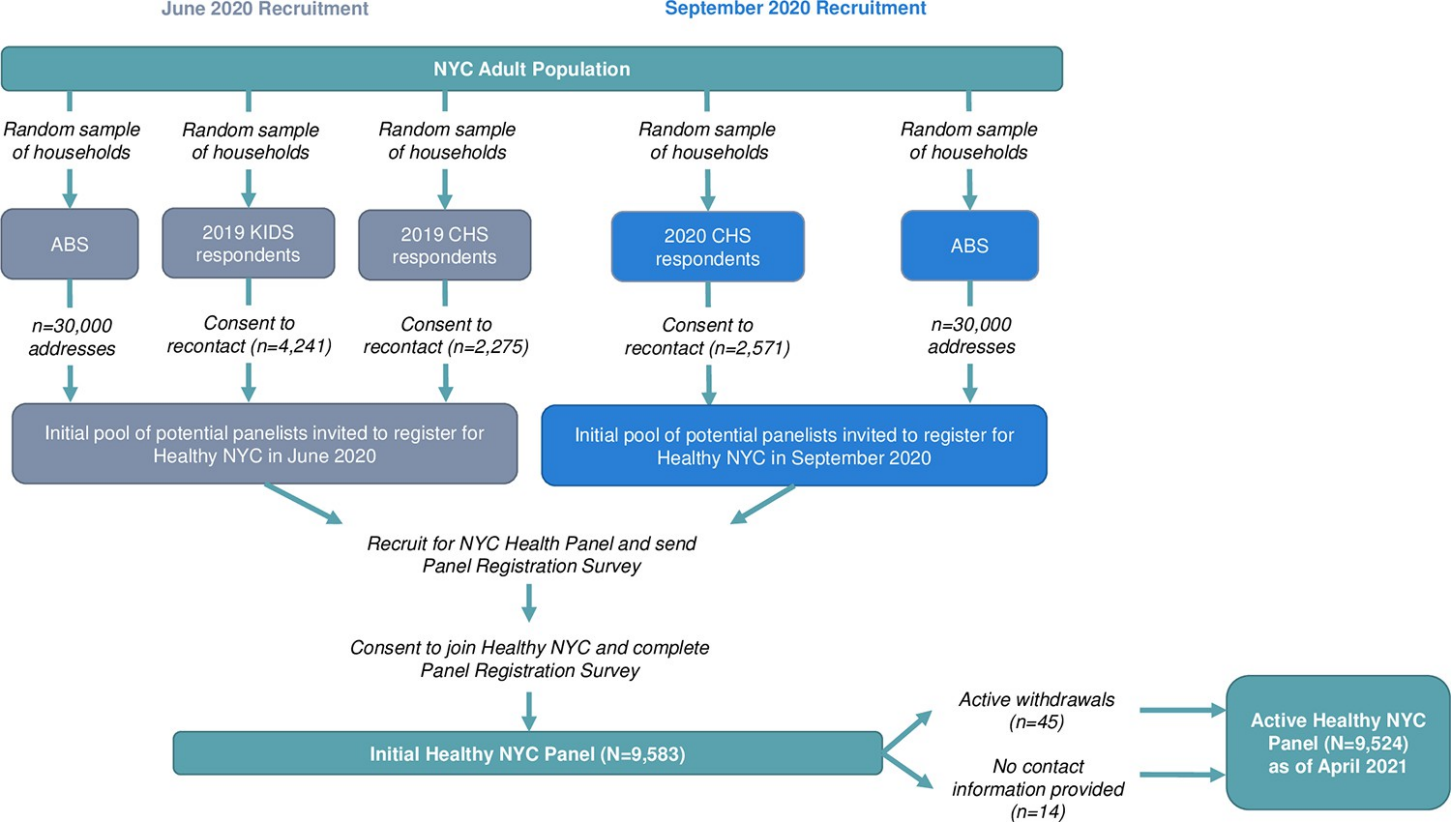
Process of recruiting NYC residents to Healthy NYC panel.

**Fig 2:**
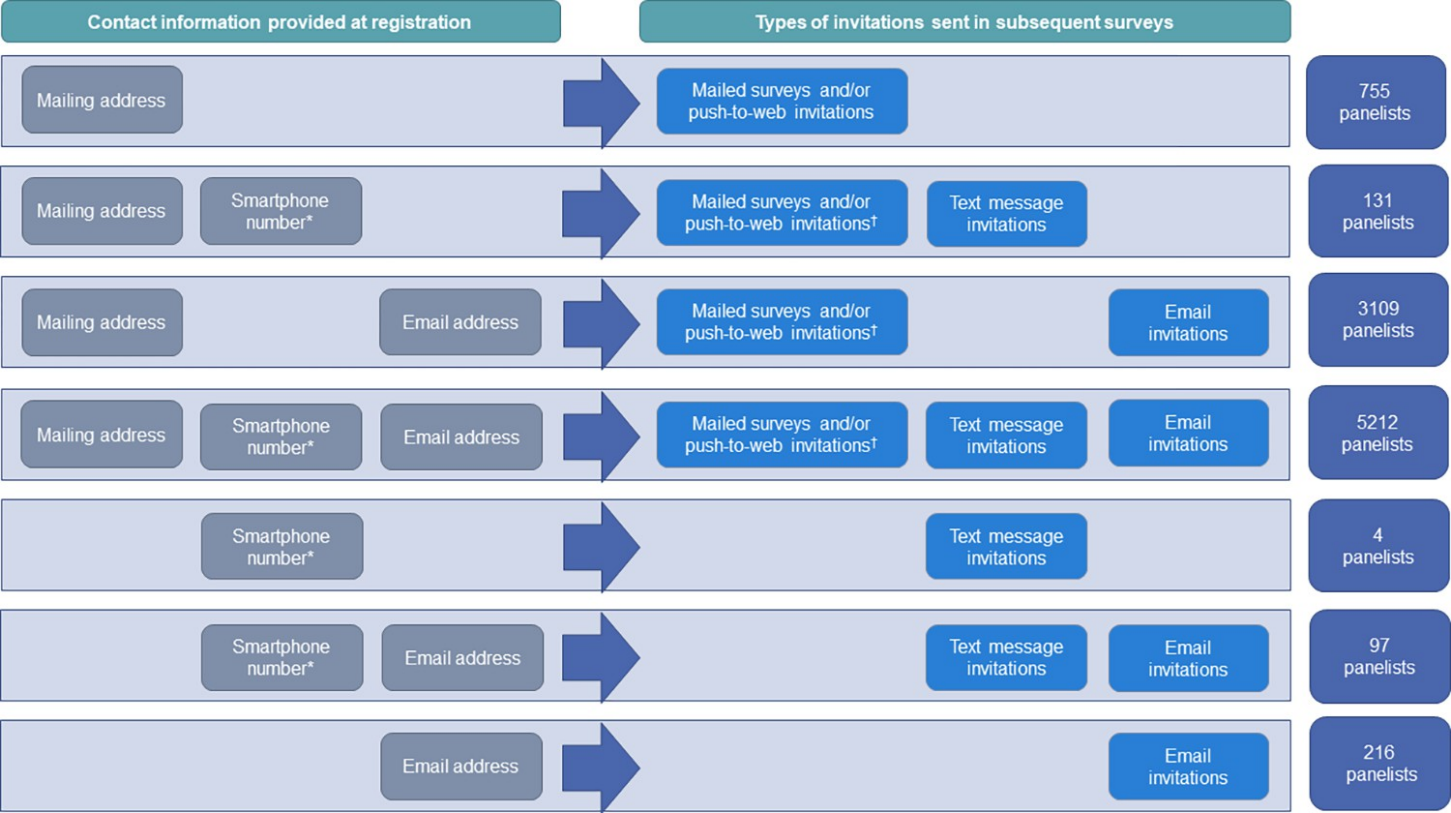
Types of contact information provided by panelists and types of invitations sent to panelists. The types of initial invitations and reminders that sampled panelists received for any given survey were determined by the types of contact information they provided when they registered for Healthy NYC. *Panelists who provided a cellphone number were asked if this was a smartphone that they could use to take surveys and were then asked to consent to receive text messages. ^†^Due to budgetary and operational limitations, not all panelists who provided a mailing address received mailed paper surveys or push-to-web invitations if they provided any other contact information.

For the first recruitment effort (6/8/2020–7/30/2020), residents from a total of 30,000 NYC addresses, and 6,516 people who had taken a previous DOHMH survey and provided a phone number and/or email address were invited to participate in Healthy NYC (Fig 1). In the second recruitment effort (09/01/2020–12/8/2020), residents from 30,000 NYC addresses and 2,571 people who had taken a previous DOHMH survey and provided a phone number and/or email address were invited to participate in Healthy NYC (Fig 1). Panelists could provide an address for mail, an email address for email contact, and a cell phone number and consent to receive text messages in the Registration survey. Fig 2 shows the frequencies and the different combinations of the three contact modes that panelists provided. At the time of the analysis (8/13/2021), a total of 9,524 panelists remained from the 9,583 initially registered after the removal of 45 withdrawals and 14 individuals who did not provide any contact information at registration.

Once enrolled, panelists can be invited to up to 10 surveys yearly. We included six surveys in this analysis: Social Determinants of Health (SDH), Emotional Wellness Survey (EWS), two waves of Health Opinion Polls (HOP), and two NYC Population Health Surveys for COVID-19 (COVID). For each survey, stratified random sampling was used to select a sample from Healthy NYC. Sampled panelists were invited by various combinations of postal mail, email, or text messages, depending on the types of contact information they provided at registration (Fig 2), and we called this the invitation mode(s).

Panelists were recruited into Healthy NYC in two waves, in June 2020 and September 2020. In both waves, most were recruited from a random Address Based Sample (ABS), supplemented by individuals who had completed previous DOHMH surveys and consented to be recontacted for future surveys. Those who completed the Registration survey were considered to be enrolled in the panel.

The surveys were self-administered online or by completing a mailed paper questionnaire. Due to funding limitations, not all panelists who provided a mailing address received a mailed invitation or a paper survey. Instead, panelists were divided into age tertiles (oldest, older, and youngest); those in the oldest category and those who did not provide an email or phone contact (that is, only provided a mailing address) were prioritized to receive mailed invitations. They first received one or two push-to-web invitations, and non-respondents received a paper survey in the final mailing. The surveys were offered in English, Spanish, Simplified and Traditional Chinese (Mandarin and Cantonese by phone), and Russian. Panelists were offered a $20 gift card for completing the June or September registration surveys and $5 for all other surveys.

There were 8,802 unique panelists sampled at least once across the six surveys; each was sampled up to five times. A total of 4,729 unique panelists responded to at least one survey.

### Sociodemographic variables

Seven sociodemographic variables were used in the analyses: borough of residence, sex assigned at birth, age, race/ethnicity, education, household income, and nativity status. Sex assigned at birth was categorized into male or female; those who answered something other than male or female were excluded from comparative analyses since the small number did not allow for meaningful comparison (<1%). Age was categorized into four groups (18–24, 25–44, 45–64, and 65+). For race/ethnicity, we combined questions on self-reported race and Latino ethnicity into the following categories: White, Black, Latino, Asian or Pacific Islander (API), and American Indian /Multiple/Something Else (AIMSE). Latino includes people of Hispanic or Latino origin, as identified by the survey question “Are you Hispanic or Latino/a?”. Those who identified as Latino/a were excluded from the other race categories. Educational attainment was categorized into four levels (less than high school, high school graduate, some college, and college graduate or higher). Household income level was defined using household Federal Poverty Level (FPL) thresholds of <200% and ≥200%. Nativity was classified as being born in the U.S. or U.S. territories or being born outside of the U.S. Missing values were excluded from the analyses.

### Statistical analysis

To determine whether panelists varied based on their sociodemographic characteristics and the contact information they provided as well as their response to the type of invitations (paper surveys, push-to-web, email, or text), we used Chi-Square tests to identify bivariate associations. This determined potential predictors for the final logistic regression models (LRM). For the contact mode analysis, there were three outcomes of interest: whether panelists provided an email address (yes/no), a mailing address (yes/no), and a smartphone number with consent to receiving text messages (yes/no).

For the analysis of which invitation mode led to a response, there were five outcomes of interest where panelists responded to: a mailed paper survey, a mailed push-to-web letter, an email, a text, or multiple invitation modes (if panelists responded to different modes of invites across surveys). The analysis for each LRM was limited to panelists who were invited at least once by the specific invitation mode. Furthermore, each LRM was restricted to panelists who were invited by at least one additional mode other than the one that led to their response.

To determine whether the number of invitations received before participating in a survey was associated with sociodemographic characteristics, we ran Poisson regression models. The dependent variable was the total number of invitations that each respondent received for each of the six surveys before they responded, and the independent variables were sociodemographic variables. Since panelists who received mail invitations received more invitations regardless of their response status, we did an additional subgroup analysis excluding panelists who received a mail invite. We did not include invitees who never responded to a survey for either analysis. Statistical significance was defined as *p*<0.05. We assessed whether there was multicollinearity between the seven sociodemographic variables. No two variables were multicollinear based on the variance inflation factor and tolerance diagnostic criteria. The final LRM and the chosen predictors were based on the lowest Akaike Information Criterion score and best Receiver Operating Characteristic curve to minimize model overfitting. Analyses were done using SAS Enterprise Guide 7.1.

### Ethics statement

The protocol for Healthy NYC was approved by the NYC DOHMH Institutional Review Board (IRB). When participants enrolled in Healthy NYC and at the start of each subsequent survey, there was consent language explaining the survey, confidentiality, and privacy protections, that participation was voluntary and that surveys could be stopped at any time. We received a waiver of documentation of informed consent from the NYC Health Department’s IRB because the research was of minimal risk and written consent was not logistically feasible. Instead, participants’ consent was documented in the following three ways: by checking a box on the web survey, by completing a paper survey, or verbally on the phone with documentation by the interviewer.

The data underlying the findings can be accessed through a Data Use Agreement (DUA) with the NYC DOHMH. Readers interested in accessing the data should contact EpiDataRequest@health.nyc.gov to engage in a DUA and receive the data.

## Results

The sociodemographic characteristics of Healthy NYC panelists are described in Table 1.

**Table 1:** Sociodemographic characteristics of Healthy NYC panelists as of June 2021 vs. sampled panelists among the six surveys.

Demographic Characteristic^1^	Healthy NYC (N = 9583)		Six Surveys (n = 8802)	
	Frequency	Percent	Frequency	Percent
**Borough**	**(n = 9582)**		**(n = 8801)**	
Bronx	1267	13.22	1208	13.73
Brooklyn	2686	28.03	2513	28.55
Manhattan	2891	30.17	2527	28.71
Queens	2240	23.38	2088	23.72
Staten Island	498	5.20	465	5.28
**Sex at Birth**	**(n = 9517)**		**(n = 8758)**	
Male	3573	37.54	3272	37.36
Female	5944	62.46	5486	62.64
**Age Group**	**(n = 9553)**		**(n = 8878)**	
18–24	468	4.90	444	5.06
25–44	4197	43.93	3952	45.02
45–64	2972	31.11	2728	31.08
65+	1916	20.06	1654	18.84
**Race/Ethnicity**	**(n = 9269)**		**(n = 8514)**	
White	4178	45.07	3796	44.59
Black	1403	15.14	1284	15.08
Latino	1960	21.15	1815	21.32
Asian/Pacific Islander	1420	15.32	1336	15.69
AIMSE^**§**^	308	3.32	283	3.32
**Education Level**	**(n = 9555)**		**(n = 8790)**	
Less than High School	684	7.16	616	7.01
High School Graduate	1236	12.94	1146	13.04
Some College	1764	18.46	1626	18.50
College Graduate	5871	61.44	5402	61.46
**Federal Poverty Level (FPL)**	**(n = 8639)**		**(n = 7988)**	
<200% FPL	2685	31.08	2541	31.81
≥200% FPL	5954	68.92	5447	68.19
**Nativity Status**	**(n = 9555)**		**(n = 8790)**	
US Born	6361	66.57	5840	66.44
Foreign Born	3194	33.43	2950	33.56

^1^ Demographic characteristics may be missing for some panelists. The number (n) denoted in parenthesis reflect the total number of panelists excluding those missing.

^**§**^ American Indian/Multiple/Something Else.

Among the 9,583 panelists, 9,250 (96.5%) provided a mailing address, 8,672 (90.5%) provided an email address, and 5,447 (56.8%) provided a smartphone number and consented to receive texts. We found that certain sociodemographic characteristics predicted the likelihood of providing a mailing address, smartphone number, or email address. Younger age, higher household income, and higher educational attainment were positively associated with providing an email address (Table 2), and younger age and higher educational attainment were positively associated with providing a smartphone number and permission to text (Table 3).

**Table 2:** Odds of providing an email contact at the time of Healthy NYC enrollment (n = 7,709)**.

Demographic Characteristic	Odds Ratio	95% CI	*p*-value
**Borough**			
Bronx*	0.72	0.53–0.98	0.04
Brooklyn*	0.71	0.54–0.92	0.01
Manhattan	reference	-	-
Queens	0.91	0.69–1.22	0.54
Staten Island	0.90	0.57–1.41	0.64
**Sex at Birth**			
Male	1.01	0.83–1.23	0.95
Female	reference	-	-
**Age Group**			
18–24	1.26	0.61–2.59	0.53
25–44	reference	-	**-**
45–64*	0.42	0.32–0.56	< .0001
65+*	0.11	0.09–0.14	< .0001
**Race/Ethnicity**			
White	reference	**-**	-
Black	0.85	0.65–1.12	0.24
Latino	1.30	0.97–1.73	0.08
Asian/Pacific Islander	1.35	0.93–1.94	0.11
AIMSE^**§**^	0.86	0.51–1.44	0.57
**Education Level**			
Less than High School	reference	-	-
High School Graduate	1.34	0.99–1.81	0.058
Some College*	3.34	2.40–4.63	< .0001
College Graduate*	4.49	3.25–6.20	< .0001
**Federal Poverty Level (FPL)**			
<200% FPL	reference	-	-
≥200% FPL*	2.15	1.73–2.68	< .0001
**Nativity Status**			
US Born	reference	**-**	-
Foreign Born	0.99	0.79–1.23	0.92

* Significant at *p* < .05.

** In the LR model, 9,583 observations were read, but 7,709 observations were used because panelists may not have provided certain sociodemographic information.

^**§**^ American Indian/Multiple/Something Else.

**Table 3:** Odds of providing a text contact at the time of Healthy NYC enrollment (n = 8,367)**.

Demographic Characteristic	Odds Ratio	95% CI	*p*-value
**Borough**			
Bronx	1.05	0.89–1.23	0.59
Brooklyn	0.94	0.84–1.07	0.35
Manhattan	reference	-	**-**
Queens	0.93	0.82–1.06	0.26
Staten Island	1.16	0.92–1.45	0.20
**Sex at Birth**			
Male	0.99	0.90–1.09	0.81
Female	reference	-	-
**Age Group**			
18–24	1.08	0.85–1.37	0.55
25–44	reference	-	**-**
45–64*	0.67	0.60–0.74	< .0001
65+*	0.29	0.25–0.33	< .0001
**Race/Ethnicity**			
White	reference	**-**	-
Black	1.16	0.99–1.35	0.05
Latino	1.17	1.01–1.34	0.03
Asian/Pacific Islander	0.91	0.79–1.06	0.24
AIMSE^**§**^	1.04	0.80–1.35	0.78
**Education Level**			
Less than High School	reference	-	-
High School Graduate	1.24	0.99–1.56	0.06
Some College*	1.64	1.32–2.03	< .0001
College Graduate*	1.74	1.40–2.15	< .0001
**Federal Poverty Level (FPL)**			
<200% FPL	reference	-	-
≥200% FPL	1.12	0.99–1.26	0.06
**Nativity Status**			
US Born	reference	-	-
Foreign Born	0.97	0.87–1.08	0.55

* Significant at *p* < .05.

** In the LR model, 9,583 observations were read, but 8,367 observations were used.

^**§**^ American Indian/Multiple/Something Else.

Furthermore, residents of both Brooklyn and Bronx had approximately 30% lesser odds of providing an email contact (OR: 0.71, 95% CI 0.0.54–0.92 and OR: 0.72, 95% CI: 0.53–0.98, respectively) compared to Manhattan residents (Table 3). Lastly, older age was positively associated with providing a mailing address (Table 4).

**Table 4:** Odds of providing a mailing address at the time of Healthy NYC enrollment (n = 8,110)^1^^,^ **.

Demographic Characteristic	Odds Ratio	95% CI	*p*-value
**Age Group**			
18–24*	0.63	0.41–0.97	0.03
25–44	reference	-	**-**
45–64*	1.89	1.41–2.52	< .0001
65+*	3.38	2.17–5.23	< .0001

^1^ Age was the only predictor that was kept in the final LRM to determine the likelihood of providing a mailing address. Predictors of the final model were based on the best fit line as assessed by ROC and lowest AIC.

* Significant at *p* < .05.

** In the LR model, 9,583 observations were read, but 8,110 observations were used.

**Fig 3:**
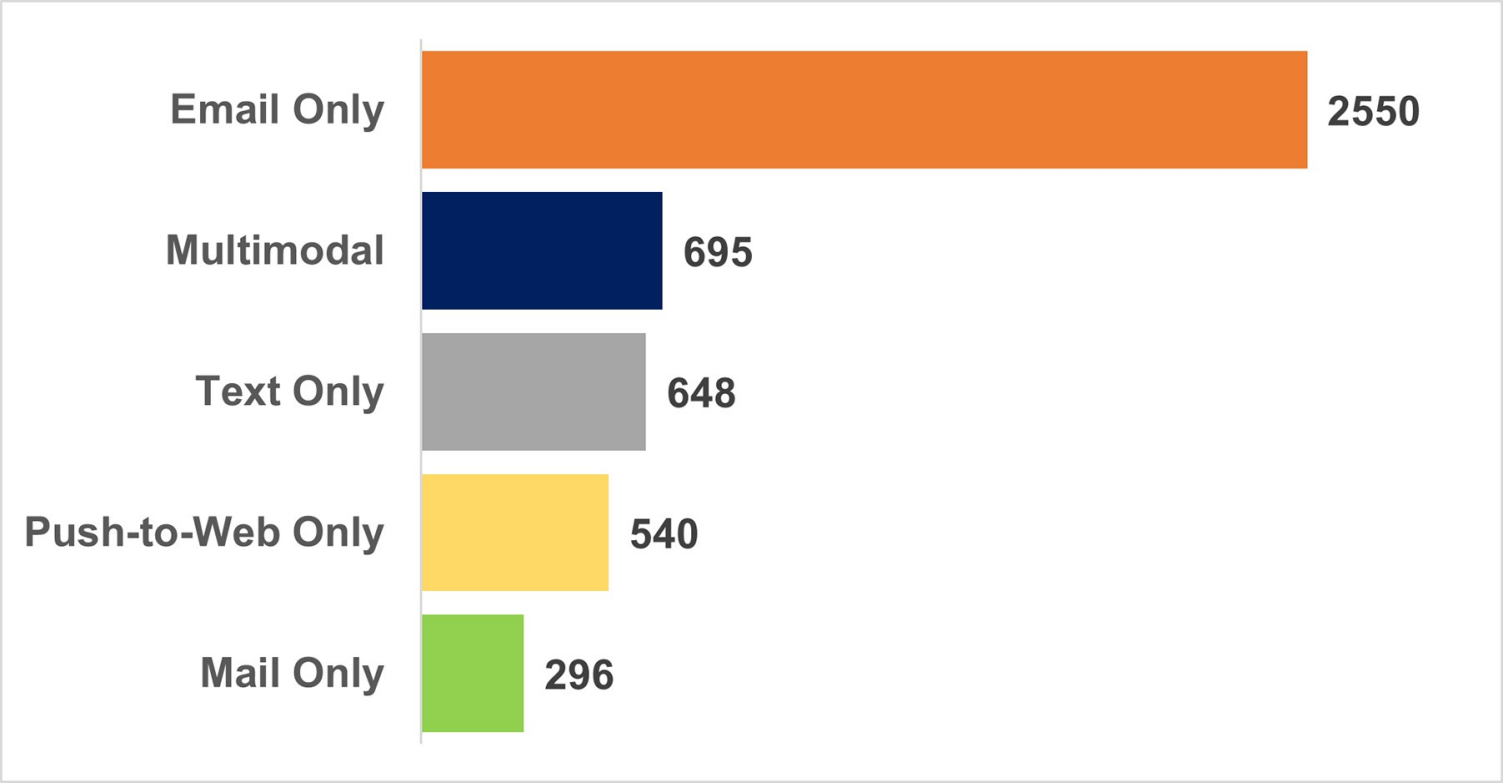
Invite mode that led to response across the six surveys (n = 4,729).

Among the 4,729 respondents who completed one or more surveys, 4,419 were invited by email, 2,413 were invited by text, 1,150 were invited by mail and sent a paper survey with a push-to-web option, 1,674 received a mail invite with only a push-to-web option (no paper survey), and 3,442 received multiple invitation modes. We found that 85% of survey participants responded exclusively to a single invite mode across the six surveys despite most (73%) receiving invitations via multiple modes. The greatest proportion of panelists (54%) responded exclusively to email invitations (Fig 3).

Age, race, and household income significantly predicted the specific invitation modes that led to the panelists’ survey responses (Tables 5–8). The odds of responding to an email invite were 52% greater for panelists ages 18–24 compared to panelists ages 25–44 (OR: 1.52, 95% CI: 1.04–2.22), but lower for panelists 45–64 and 65+ compared to panelists ages 25–44 (OR: 0.66, 95% CI 0.57–0.77 and OR: 0.18, 95% CI: 0.15–0.22, respectively). Furthermore, the odds of responding to text invites were 84% lower for panelists ages 65+ compared to panelists ages 25–44 (OR: 0.16, 95% CI: 0.10–0.26); however, no differences were seen between panelists ages 18–24 and 25–44.

**Table 5:** Odds of responding to an email invitation^1^.

Demographic Characteristic	Odds Ratio	95% CI	*p*-value
**Borough**			
Bronx*	0.74	0.59–0.94	0.01
Brooklyn*	0.82	0.69–0.98	0.03
Manhattan	reference	-	-
Queens*	0.81	0.67–0.98	0.03
Staten Island	0.73	0.53–1.00	0.05
**Age Group**			
18–24*	1.52	1.04–2.22	0.03
25–44	reference	-	-
45–64*	0.66	0.57–0.77	< .0001
65+*	0.18	0.15–0.22	< .0001
**Race/Ethnicity**			
White	reference	**-**	-
Black	0.67	0.54–0.83	< .0001
Latino	0.85	0.70–1.03	0.09
Asian/Pacific Islander	1.33	1.08–1.64	0.01
AIMSE^**§**^	0.71	0.48–1.06	0.09

^1^ Predictors of the final model were based on the best fit line as assessed by ROC and lowest AIC.

* Significant at *p* < .05.

^**§**^ American Indian/Multiple/Something Else.

**Table 6:** Odds of responding to a text invitation^1^.

Demographic Characteristic	Odds Ratio	95% CI	*p*-value
**Age Group**			
18–24	0.66	0.41–1.07	0.09
25–44	reference	-	-
45–64	0.91	0.74–1.12	0.37
65+*	0.16	0.10–0.26	<0.0001
**Education Level**			
Less than High School	reference	-	-
High School Graduate*	0.40	0.24–0.69	0.0009
Some College*	0.46	0.28–0.74	0.001
College Graduate*	0.57	0.36–0.88	0.01

^1^ Predictors of the final model were based on the best fit line as assessed by ROC and lowest AIC.

* Significant at *p* < .05.

**Table 7:** Odds of responding to a mail (paper survey) invitation^1^.

Demographic Characteristic	Odds Ratio	95% CI	*p*-value
**Borough**			
Bronx	1.22	0.70–2.10	0.35
Brooklyn	1.36	0.85–2.19	0.20
Manhattan	reference	-	-
Queens	1.26	0.77–2.07	0.48
Staten Island	1.52	0.70–3.28	0.29
**Sex at Birth**			
Male	0.99	0.70–1.39	0.95
Female	reference	-	-
**Age Group**			
18–24^a^	-	-	-
25–44	reference	-	-
45–64	0.39	0.10–1.55	0.18
65+	0.79	0.20–3.09	0.73
**Race/Ethnicity**			
White	reference	**-**	-
Black*	1.72	1.11–2.68	0.02
Latino	0.65	0.37–1.15	0.16
Asian/Pacific Islander	1.06	0.55–2.01	0.87
AIMSE*^§^	3.25	1.27–8.35	0.01
**Education Level**			
Less than High School	reference	-	**-**
High School Graduate	1.59	0.82–3.05	0.16
Some College	0.74	0.38–1.42	0.36
College Graduate	0.61	0.32–1.17	0.13
**Federal Poverty Level (FPL)**			
<200% FPL	reference	-	-
≥200% FPL*	0.47	0.32–0.68	< .0001

^1^ Predictors of the final model were based on the best fit line as assessed by ROC and lowest AIC.

^a^ Panelists in the 18–24 age group were not sent paper surveys. Paper surveys were prioritized for older age groups.

^**§**^ American Indian/Multiple/Something Else.

* Significant at *p* < .05.

**Table 8:** Odds of responding to a mail (push-to-web) letter invitation^1^^,^ ^2^.

Demographic Characteristic	Odds Ratio	95% CI	*p*-value
**Age Group**			
18–24^a^	-	-	-
25–44	reference	-	-
45–64	0.51	0.19–1.34	0.17
65+	0.98	0.37–2.57	0.97
**Federal Poverty Level (FPL)**			
<200% FPL	reference	-	-
≥200% FPL*	2.24	1.74–2.87	< .0001

^1^ The final model was based on the best fit line as assessed by ROC and lowest AIC.

^2^ Age and FPL were the only predictors kept in the final LRM to determine the likelihood of providing a push-to-web contact.

^a^ Some panelists in the 18–24 age group were sent mailed push-to-web invitations, but those panelists were not invited by any other invitation modes. Thus, they were excluded from this analysis since the LRM was restricted to panelists who were invited by at least one additional mode other than the one that led to their response.

* Significant at *p* < .05.

Race/ethnicity was significantly associated with responding to an email or paper survey. The odds of responding to an email invite were 33% greater for API panelists (OR: 1.33, 95% CI: 1.08–1.64) and 33% less for Black panelists (OR 0.67, 95% CI:0.54–0.83) compared to White panelists. Black panelists had 72% greater odds of responding to a mailed paper survey compared to White panelists (OR 1.72 95% CI: 1.11–2.68).

Those with higher household income had 53% lesser odds of responding to a paper survey (OR: 0.47 95% CI: 0.32–0.68) and 124% greater odds of responding to a push-to-web letter invite (OR: 2.24, 95% CI: 1.74–2.87) compared to those with lower household income.

**Fig 4:**
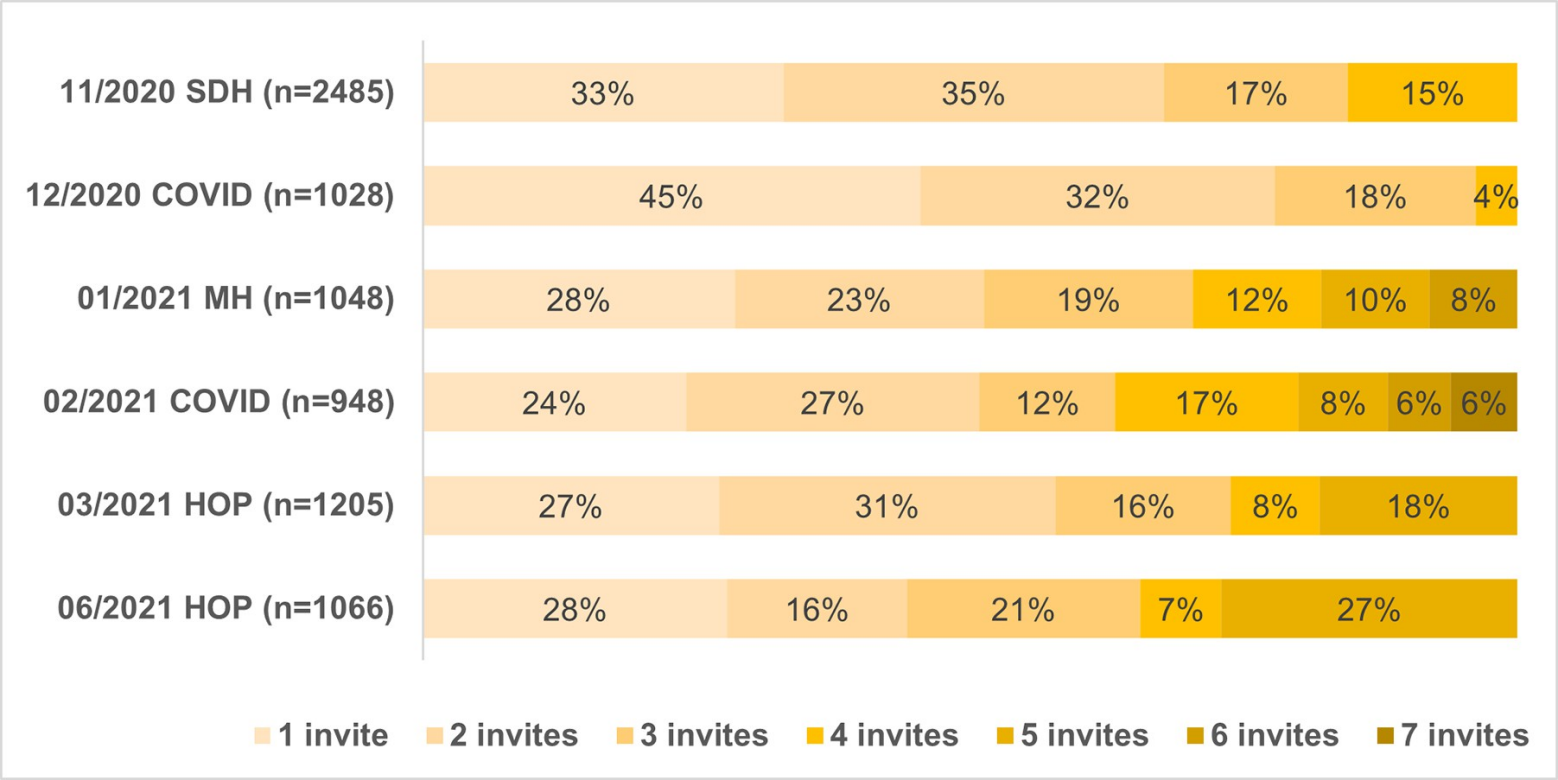
Number of invites participants received before response (by the total number of respondents for that survey).

Finally, across the six surveys, panelists received between one and seven invites for any given survey (Fig 4). In five of the six surveys, more than half of the respondents completed surveys after one or two invites, except for the June HOP.

**Fig 5:**
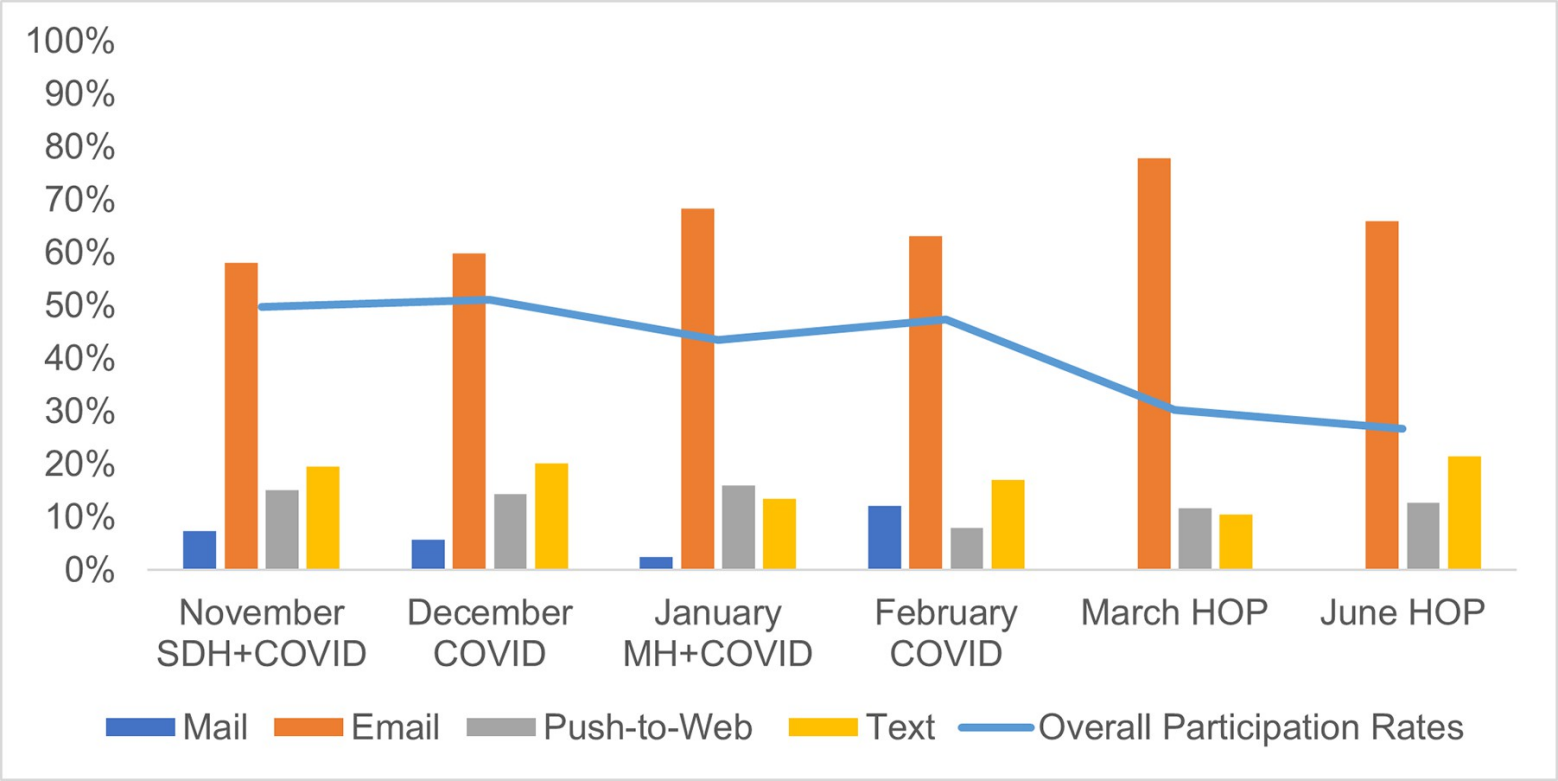
Percentage of invite mode that led to responses by month with overall response rate.

Email invitations resulted in the highest number of responses (Fig 5). The participation rate shows a slight decline over this time-period. However, it should be noted that March and June HOP had a two-week data collection period, as opposed to four weeks for other survey months. There was no association between panelists’ sociodemographic characteristics and the number of invitations they received before participating in a survey (Table 9 and S1 Table). In the Poisson models that include all respondents, age was associated with the number of invitations sent before eliciting a response. Across all six surveys, respondents 65+ were sent significantly more invitations before responding compared to those ages 25–44, and respondents 45–64 were sent significantly more invitations compared to those ages 25–44 in four out of six surveys. However, when respondents invited by mail were excluded from the analysis, age was no longer a significant predictor of the number of invitations sent before eliciting a response to most surveys (Table 9 and S1 Table).

**Table 9:** Poisson regression table of age with number of invitations received before participating in a survey (including and excluding mail invitations).

**Survey Month and Age Group**	**Poisson Analysis Including Mail Invitations (Email, Text, and Mail)**				**Poisson Analysis Excluding Mail Invitations (Email and Text Only)**			
	**Odds Ratio**	**95% Lower Confidence Limit**	**95% Upper Confidence Limit**	***p*-value**	**Odds Ratio**	**95% Lower Confidence Limit**	**95% Upper Confidence Limit**	***p*-value**
**NOVEMBER SDH**								
18–24	0.91	0.79	1.06	0.22	0.91	0.79	1.06	0.23
25–44	1.00	1.00	1.00	.	1.00	1.00	1.00	.
45–64	1.30	1.22	1.38	<0.01	0.96	0.89	1.03	0.25
65+	1.61	1.52	1.71	<0.01	0.54	0.37	0.79	<0.01
**DECEMBER COVID**								
18–24	0.95	0.79	1.16	0.63	0.96	0.79	0.96	0.64
25–44	1.00	1.00	1.00	.	1.00	1.00	1.00	.
45–64	1.41	1.28	1.54	<0.01	1.13	0.99	1.13	0.06
65+**	1.52	1.36	1.69	<0.01	--	--	--	--
**JANUARY MH**								
18–24	0.80	0.64	1.00	0.05	0.80	0.64	1.00	0.05
25–44	1.00	1.00	1.00	.	1.00	1.00	1.00	.
45–64	1.30	1.19	1.41	<0.01	1.08	0.97	1.21	0.15
65+	1.16	1.05	1.29	<0.01	0.79	0.49	1.27	0.33
**Survey Month and Age Group**	**Poisson Analysis Including Mail Invitations (Email, Text, and Mail)**				**Poisson Analysis Excluding Mail Invitations (Email and Text Only)**			
	**Odds Ratio**	**95% Lower Confidence Limit**	**95% Upper Confidence Limit**	***p*-value**	**Odds Ratio**	**95% Lower Confidence Limit**	**95% Upper Confidence Limit**	***p*-value**
**FEBRUARY COVID**								
18–24	0.84	0.65	1.09	0.19	0.84	0.65	1.09	0.19
25–44	1.00	1.00	1.00	.	1.00	1.00	1.00	.
45–64	1.11	1.00	1.23	0.05	1.02	0.91	1.14	0.72
65+	1.53	1.39	1.69	<0.01	1.28	0.58	2.87	0.54
**MARCH HOP**								
18–24	0.97	0.78	1.21	0.79	0.97	0.78	1.22	0.81
25–44	1.00	1.00	1.00	.	1.00	1.00	1.00	.
45–64	1.20	1.08	1.33	<0.01	1.05	0.93	1.18	0.47
65+	1.47	1.31	1.66	<0.01	1.16	0.86	1.56	0.33
**JUNE HOP**								
18–24	0.91	0.72	1.14	0.41	0.91	0.72	1.14	0.41
25–44	1.00	1.00	1.00	.	1.00	1.00	1.00	.
45–64	1.10	0.99	1.22	0.09	1.02	0.91	1.15	0.69
65+	1.48	1.33	1.64	<0.01	1.30	0.54	3.14	0.55

** For the December COVID survey, there too few panelists in the 65+ age category who received exclusively email or text invites and, therefore, there were insufficient data points to perform the analysis.

## Discussion

In our six surveys, we found that certain sociodemographic variables predicted panelists’ likelihood of providing certain types of contact information. Additionally, some sociodemographic variables predicted panelists’ likelihood of responding to a survey invitations received by mail, text, or email. However, sociodemographic variables did not predict the number of invitations needed in order to provide a survey response. Based on our findings, it is imperative to offer mixed-mode survey invitations to ensure panelist engagement and participation among respondents with a range of sociodemographic characteristics; this finding is consistent with past studies [22–25].

An important issue in survey research is ensuring representation from all population groups, including those who are traditionally more difficult to reach in surveys [26, 27]. Strategies, such as oversampling and using non-probability frames, have been cited as potential solutions [28–30]. Although there is research on using various targeted invitation designs to improve survey participation [31–33], our findings can help other researchers develop an invitation strategy tailored to the subpopulations being surveyed in panels and identify response patterns. For example, past studies have found that Black survey invitees are more likely to complete surveys by mail compared to the web, as our findings corroborate [34, 35]. We recommend using targeted invitation modes as an additional strategy to improve participation when needed. Yet, it is noteworthy that 85% of our active panelists consistently responded to the same type of invitation and may, therefore, only require that specific type of invitation in the future.

In our study, panelists with lower household incomes were more likely to respond to a mailed paper survey and less likely to respond to a push-to-web invite than panelists with higher-income households. Even though internet access and usage are widespread among the general population, inequities surrounding internet access remain. Some panelists may have limited or no access and, therefore, may not be able to complete a survey online. Approximately 12.4% of NYC households do not have internet access [36], and neighborhoods with a higher proportion of racially and ethnically marginalized groups, households with lower household income, higher poverty levels, and residents with lower educational attainment have more limited internet access [37]. Although there was no difference between panelists with different household incomes in their propensity to respond to an email invite, we found that panelists with higher household incomes were much more likely to provide an email contact compared to panelists with lower household incomes. Therefore, it may be reasonable to provide lower-income panelists who do not provide an email contact with additional response options not requiring internet access (for example, phone or paper).

Lastly, we found that the number of invitations sent before receiving a response is likely not predicted by sociodemographic characteristics. In the analyses that included mail invitations, panelists who were 45–64 or 65+ years old were sent more invitations before responding across most or all of the six surveys. However, once mailed invitations were excluded from the analysis, there was no longer a consistent association between age and the number of invitations required to elicit a response. This difference in the findings can be attributed to three issues related to the design of the survey administration.

First, older panelists were prioritized to receive mailed push-to-web and follow-up paper surveys based on the assumption that older adults would be less likely to respond to email and text invitations [35, 38–40]. Second, since the push-to-web and paper surveys were distributed by a vendor that required up to two weeks to prepare, print, and mail the materials, there was limited opportunity to remove individuals from reminder mailing lists once they responded to the survey. Therefore, unlike with web responses, we were unable to remove persons who responded to a mail invite from receiving future paper invitations so these people could have received an invitation after survey completion. Third, participants were told that they would receive a paper survey in the mail towards the end of the fielding period if needed; some panelists may have waited to receive the paper survey to respond. In summary, since recipients of push-to-web and mailed paper surveys were sent at least two invites regardless of response status and may have waited to get a paper survey, they likely received more invitations because of this operational arrangement.

Our study has some limitations. This analysis includes only six survey months and analyzing additional surveys across a longer period can further validate the findings. Furthermore, in an effort to balance both budgetary constraints and operational needs, especially during the COVID-19 pandemic, we were not able to randomize the types of invitations that panelists received. A randomized experiment that included only panelists who provided all three types of contact information would have allowed us to draw stronger inferences about the association between panelists’ sociodemographic characteristics and their likelihood of responding to various types of invitations. Instead, we included individuals who provided at least one type of contact information and we sent invitations based on the contact modes panelists provided, and the limited mailed invitations were prioritized to older panelists and those who provided only a mailing address for contact. Past research has shown that older survey respondents are more likely to respond to mailed paper surveys than online surveys [35, 38–40]. These studies corroborate our findings that–even among those who provided email and/or text contact information–older panelists were less likely to respond to email and text invitations than younger panelists.

Strategies to maintain an engaged panel that is representative of the NYC population are crucial in improving the accuracy of public health surveillance data. The data collected through Healthy NYC provide guidance to public health leaders for developing evidence-based policies and programs to improve the health and wellness of NYC residents, and it is essential that these data are representative of the population being served. As the Healthy NYC panel matures, we plan to increase representativeness of each survey by targeting invitations based on panelists’ preferences, as described in survey questions about their preferred way to be contacted, as well as past response patterns. We will also refine our invitation approaches by assessing whether certain combinations of invitation modes lead to better participation rates within certain sociodemographic subgroups of the panel. For example, an initial email invitation followed by a text reminder may be more motivating for some populations than an initial text invitation followed by an email reminder.

In conclusion, this research provides valuable insight into a diverse probability-based survey panel and the potential benefits of using a targeted invitation strategy. In a city agency with limited resources, improving survey response through targeted invitations in panels can be cost-effective and efficient.

## Supporting information

S1 Table Raw tables of the six poisson regression models (mail invitations included). (DOCX)

S2 Table Raw tables of the six poisson regression models (email and text invitations only). (DOCX)
