# Supplementary material for: Assessing targeted invitation and response modes to improve survey participation in a diverse New York City panel: Healthy NYC
Source: PLoS One. 2023 Jan 26;18(1):e0280911. doi: 10.1371/journal.pone.0280911 (PMC9879422; doi:10.1371/journal.pone.0280911)
Supplement: S1 Table — (DOCX) [file pone.0280911.s001.docx]

# **Supporting Information**

| **S1 Table: Raw Tables of the Six Poisson Regression Models (Mail Invitations Included)** | | | | | | | |
| --- | --- | --- | --- | --- | --- | --- | --- |
| **Demographic Characteristics** | **DF** | **Estimate** | **Standard Error** | **95% Lower Confidence Limit** | **95% Upper Confidence Limit** | **Wald Chi-Square** | **Pr > ChiSq** |
| **NOVEMBER SDH** | | | | | | | |
| Intercept | 1 | 0.8521 | 0.0772 | 0.7008 | 1.0034 | 121.86 | <.0001 |
| 18-24 | 1 | -0.0867 | 0.0763 | -0.2363 | 0.0630 | 1.29 | 0.2563 |
| 25-44 | 0 | 0.0000 | 0.0000 | 0.0000 | 0.0000 | . | . |
| 45-64 | 1 | 0.2419 | 0.0305 | 0.1821 | 0.3018 | 62.81 | <.0001 |
| 65+ | 1 | 0.4677 | 0.0327 | 0.4037 | 0.5318 | 204.92 | <.0001 |
| Less than high school | 0 | 0.0000 | 0.0000 | 0.0000 | 0.0000 | . | . |
| High school graduate | 1 | -0.0103 | 0.0683 | -0.1442 | 0.1236 | 0.02 | 0.8798 |
| Some college | 1 | 0.0272 | 0.0654 | -0.1009 | 0.1553 | 0.17 | 0.6772 |
| College graduate | 1 | -0.0206 | 0.0644 | -0.1468 | 0.1056 | 0.10 | 0.7491 |
| <200%FPL | 1 | -0.0421 | 0.0321 | -0.1050 | 0.0208 | 1.72 | 0.1898 |
| ≥200%FPL | 0 | 0.0000 | 0.0000 | 0.0000 | 0.0000 | . | . |
| White | 0 | 0.0000 | 0.0000 | 0.0000 | 0.0000 | . | . |
| Black | 1 | 0.1190 | 0.0407 | 0.0392 | 0.1989 | 8.53 | 0.0035 |
| Hispanic | 1 | 0.0869 | 0.0398 | 0.0089 | 0.1649 | 4.77 | 0.0290 |
| Asian | 1 | -0.0282 | 0.0432 | -0.1128 | 0.0564 | 0.43 | 0.5135 |
| Other | 1 | 0.0176 | 0.0781 | -0.1354 | 0.1705 | 0.05 | 0.8221 |
| Bronx | 1 | 0.0205 | 0.0445 | -0.0666 | 0.1077 | 0.21 | 0.6443 |
| Brooklyn | 1 | -0.0214 | 0.0331 | -0.0863 | 0.0434 | 0.42 | 0.5173 |
| Queens | 1 | 0.0301 | 0.0349 | -0.0382 | 0.0985 | 0.75 | 0.3873 |
| Manhattan | 0 | 0.0000 | 0.0000 | 0.0000 | 0.0000 | . | . |
| Staten Island | 1 | 0.0450 | 0.0560 | -0.0647 | 0.1547 | 0.65 | 0.4211 |
| Male | 1 | 0.0744 | 0.0260 | 0.0234 | 0.1255 | 8.18 | 0.0042 |
| Female | 0 | 0.0000 | 0.0000 | 0.0000 | 0.0000 | . | . |
| US Born | 1 | -0.0216 | 0.0304 | -0.0811 | 0.0380 | 0.50 | 0.4780 |
| Non-US Born | 0 | 0.0000 | 0.0000 | 0.0000 | 0.0000 | . | . |

| **S1 Table: Raw Tables of the Six Poisson Regression Models (Mail Invitations Included)** | | | | | | | |
| --- | --- | --- | --- | --- | --- | --- | --- |
| **Demographic Characteristics** | **DF** | **Estimate** | **Standard Error** | **95% Lower Confidence Limit** | **95% Upper Confidence Limit** | **Wald Chi-Square** | **Pr > ChiSq** |
| **DECEMBER COVID** | | | | | | | |
| Intercept | 1 | 0.8997 | 0.1308 | 0.6433 | 1.1561 | 47.29 | <.0001 |
| 18-24 | 1 | -0.0734 | 0.1000 | -0.2694 | 0.1227 | 0.54 | 0.4633 |
| 25-44 | 0 | 0.0000 | 0.0000 | 0.0000 | 0.0000 | . | . |
| 45-64 | 1 | 0.3158 | 0.0474 | 0.2230 | 0.4086 | 44.46 | <.0001 |
| 65+ | 1 | 0.3980 | 0.0573 | 0.2858 | 0.5102 | 48.32 | <.0001 |
| Less than high school | 0 | 0.0000 | 0.0000 | 0.0000 | 0.0000 | . | . |
| High school graduate | 1 | -0.0433 | 0.1157 | -0.2701 | 0.1835 | 0.14 | 0.7080 |
| Some college | 1 | -0.0765 | 0.1128 | -0.2975 | 0.1445 | 0.46 | 0.4973 |
| College graduate | 1 | -0.1599 | 0.1093 | -0.3743 | 0.0544 | 2.14 | 0.1435 |
| <200%FPL | 1 | -0.0519 | 0.0491 | -0.1482 | 0.0444 | 1.12 | 0.2905 |
| ≥200%FPL | 0 | 0.0000 | 0.0000 | 0.0000 | 0.0000 | . | . |
| White | 0 | 0.0000 | 0.0000 | 0.0000 | 0.0000 | . | . |
| Black | 1 | 0.1195 | 0.0630 | -0.0039 | 0.2430 | 3.60 | 0.0578 |
| Hispanic | 1 | -0.0133 | 0.0631 | -0.1370 | 0.1103 | 0.04 | 0.8324 |
| Asian | 1 | 0.0042 | 0.0664 | -0.1259 | 0.1344 | 0.00 | 0.9493 |
| Other | 1 | 0.0161 | 0.1281 | -0.2351 | 0.2673 | 0.02 | 0.9001 |
| Bronx | 1 | 0.1692 | 0.0677 | 0.0364 | 0.3019 | 6.24 | 0.0125 |
| Brooklyn | 1 | 0.0135 | 0.0560 | -0.0962 | 0.1233 | 0.06 | 0.8090 |
| Manhattan | 0 | 0.0000 | 0.0000 | 0.0000 | 0.0000 | . | . |
| Queens | 1 | 0.0585 | 0.0596 | -0.0583 | 0.1753 | 0.96 | 0.3262 |
| Staten Island | 1 | 0.0663 | 0.1018 | -0.1332 | 0.2658 | 0.42 | 0.5149 |
| Male | 1 | -0.0398 | 0.0430 | -0.1241 | 0.0445 | 0.86 | 0.3549 |
| Female | 0 | 0.0000 | 0.0000 | 0.0000 | 0.0000 | . | . |
| US Born | 1 | -0.0360 | 0.0495 | -0.1330 | 0.0610 | 0.53 | 0.4668 |
| Non-US Born | 0 | 0.0000 | 0.0000 | 0.0000 | 0.0000 | . | . |

| **S1 Table: Raw Tables of the Six Poisson Regression Models (Mail Invitations Included)** | | | | | | | |
| --- | --- | --- | --- | --- | --- | --- | --- |
| **Demographic Characteristics** | **DF** | **Estimate** | **Standard Error** | **95% Lower Confidence Limit** | **95% Upper Confidence Limit** | **Wald Chi-Square** | **Pr > ChiSq** |
| **JANUARY MH** | | | | | | | |
| Intercept | 1 | 0.8514 | 0.1184 | 0.6194 | 1.0834 | 51.74 | <.0001 |
| 18-24 | 1 | -0.2552 | 0.1143 | -0.4792 | -0.0313 | 4.99 | 0.0255 |
| 25-44 | 0 | 0.0000 | 0.0000 | 0.0000 | 0.0000 | . | . |
| 45-64 | 1 | 0.2598 | 0.0437 | 0.1741 | 0.3454 | 35.30 | <.0001 |
| 65+ | 1 | 0.1555 | 0.0539 | 0.0499 | 0.2610 | 8.33 | 0.0039 |
| Less than high school | 0 | 0.0000 | 0.0000 | 0.0000 | 0.0000 | . | . |
| High school graduate | 1 | 0.0312 | 0.1089 | -0.1822 | 0.2446 | 0.08 | 0.7742 |
| Some college | 1 | 0.0424 | 0.1025 | -0.1585 | 0.2432 | 0.17 | 0.6792 |
| College graduate | 1 | 0.0329 | 0.1009 | -0.1649 | 0.2306 | 0.11 | 0.7447 |
| <200%FPL | 1 | 0.0614 | 0.0482 | -0.0331 | 0.1560 | 1.62 | 0.2029 |
| ≥200%FPL | 0 | 0.0000 | 0.0000 | 0.0000 | 0.0000 | . | . |
| White | 0 | 0.0000 | 0.0000 | 0.0000 | 0.0000 | . | . |
| Black | 1 | 0.1295 | 0.0597 | 0.0125 | 0.2465 | 4.70 | 0.0301 |
| Hispanic | 1 | -0.0284 | 0.0601 | -0.1462 | 0.0895 | 0.22 | 0.6372 |
| Asian | 1 | 0.0910 | 0.0641 | -0.0347 | 0.2167 | 2.01 | 0.1558 |
| Other | 1 | -0.0820 | 0.1229 | -0.3229 | 0.1588 | 0.45 | 0.5044 |
| Bronx | 1 | 0.1052 | 0.0661 | -0.0244 | 0.2348 | 2.53 | 0.1115 |
| Brooklyn | 1 | 0.0440 | 0.0522 | -0.0584 | 0.1464 | 0.71 | 0.3995 |
| Manhattan | 0 | 0.0000 | 0.0000 | 0.0000 | 0.0000 | . | . |
| Queens | 1 | 0.0653 | 0.0553 | -0.0430 | 0.1736 | 1.40 | 0.2375 |
| Staten Island | 1 | 0.0873 | 0.0914 | -0.0918 | 0.2664 | 0.91 | 0.3393 |
| Male | 1 | 0.0106 | 0.0396 | -0.0671 | 0.0883 | 0.07 | 0.7890 |
| Female | 0 | 0.0000 | 0.0000 | 0.0000 | 0.0000 | . | . |
| US Born | 1 | 0.0171 | 0.0465 | -0.0741 | 0.1082 | 0.13 | 0.7136 |
| Non-US Born | 0 | 0.0000 | 0.0000 | 0.0000 | 0.0000 | . | . |

| **S1 Table: Raw Tables of the Six Poisson Regression Models (Mail Invitations Included)** | | | | | | | |
| --- | --- | --- | --- | --- | --- | --- | --- |
| **Demographic Characteristics** | **DF** | **Estimate** | **Standard Error** | **95% Lower Confidence Limit** | **95% Upper Confidence Limit** | **Wald Chi-Square** | **Pr > ChiSq** |
| **FEBRUARY COVID** | | | | | | | |
| Intercept | 1 | 0.9917 | 0.1204 | 0.7558 | 1.2276 | 67.88 | <.0001 |
| 18-24 | 1 | -0.1639 | 0.1325 | -0.4236 | 0.0959 | 1.53 | 0.2162 |
| 25-44 | 0 | 0.0000 | 0.0000 | 0.0000 | 0.0000 | . | . |
| 45-64 | 1 | 0.0894 | 0.0534 | -0.0153 | 0.1940 | 2.80 | 0.0942 |
| 65+ | 1 | 0.4146 | 0.0537 | 0.3094 | 0.5197 | 59.70 | <.0001 |
| Less than high school | 0 | 0.0000 | 0.0000 | 0.0000 | 0.0000 | . | . |
| High school graduate | 1 | -0.0695 | 0.1047 | -0.2747 | 0.1357 | 0.44 | 0.5069 |
| Some college | 1 | -0.0591 | 0.0992 | -0.2536 | 0.1354 | 0.36 | 0.5512 |
| College graduate | 1 | -0.1301 | 0.0972 | -0.3206 | 0.0603 | 1.79 | 0.1806 |
| <200%FPL | 1 | -0.0842 | 0.0515 | -0.1851 | 0.0168 | 2.67 | 0.1022 |
| ≥200%FPL | 0 | 0.0000 | 0.0000 | 0.0000 | 0.0000 | . | . |
| White | 0 | 0.0000 | 0.0000 | 0.0000 | 0.0000 | . | . |
| Black | 1 | 0.0727 | 0.0649 | -0.0544 | 0.1998 | 1.26 | 0.2623 |
| Hispanic | 1 | 0.0621 | 0.0662 | -0.0677 | 0.1918 | 0.88 | 0.3484 |
| Asian | 1 | 0.0316 | 0.0707 | -0.1069 | 0.1701 | 0.20 | 0.6549 |
| Other | 1 | -0.2299 | 0.1380 | -0.5005 | 0.0406 | 2.77 | 0.0958 |
| Bronx | 1 | 0.0630 | 0.0726 | -0.0794 | 0.2053 | 0.75 | 0.3858 |
| Brooklyn | 1 | -0.0282 | 0.0580 | -0.1419 | 0.0854 | 0.24 | 0.6263 |
| Manhattan | 0 | 0.0000 | 0.0000 | 0.0000 | 0.0000 | . | . |
| Queens | 1 | -0.0228 | 0.0618 | -0.1439 | 0.0983 | 0.14 | 0.7121 |
| Staten Island | 1 | 0.0082 | 0.0993 | -0.1864 | 0.2028 | 0.01 | 0.9342 |
| Male | 1 | 0.0320 | 0.0441 | -0.0544 | 0.1184 | 0.53 | 0.4680 |
| Female | 0 | 0.0000 | 0.0000 | 0.0000 | 0.0000 | . | . |
| US Born | 1 | -0.0502 | 0.0488 | -0.1459 | 0.0455 | 1.06 | 0.3036 |
| Non-US Born | 0 | 0.0000 | 0.0000 | 0.0000 | 0.0000 | . | . |

| **S1 Table: Raw Tables of the Six Poisson Regression Models (Mail Invitations Included)** | | | | | | | |
| --- | --- | --- | --- | --- | --- | --- | --- |
| **Demographic Characteristics** | **DF** | **Estimate** | **Standard Error** | **95% Lower Confidence Limit** | **95% Upper Confidence Limit** | **Wald Chi-Square** | **Pr > ChiSq** |
| **MARCH HOP** | | | | | | | |
| Intercept | 1 | 0.9917 | 0.1204 | 0.7558 | 1.2276 | 67.88 | <.0001 |
| 18-24 | 1 | -0.1639 | 0.1325 | -0.4236 | 0.0959 | 1.53 | 0.2162 |
| 25-44 | 0 | 0.4146 | 0.0537 | 0.3094 | 0.5197 | 59.70 | <.0001 |
| 45-64 | 1 | 0.0000 | 0.0000 | 0.0000 | 0.0000 | . | . |
| 65+ | 1 | 0.0894 | 0.0534 | -0.0153 | 0.1940 | 2.80 | 0.0942 |
| Less than high school | 0 | -0.1301 | 0.0972 | -0.3206 | 0.0603 | 1.79 | 0.1806 |
| High school graduate | 1 | -0.0695 | 0.1047 | -0.2747 | 0.1357 | 0.44 | 0.5069 |
| Some College | 1 | -0.0591 | 0.0992 | -0.2536 | 0.1354 | 0.36 | 0.5512 |
| College graduate | 1 | 0.0000 | 0.0000 | 0.0000 | 0.0000 | . | . |
| <200%FPL | 1 | -0.0842 | 0.0515 | -0.1851 | 0.0168 | 2.67 | 0.1022 |
| ≥200%FPL | 0 | 0.0000 | 0.0000 | 0.0000 | 0.0000 | . | . |
| White | 0 | -0.2299 | 0.1380 | -0.5005 | 0.0406 | 2.77 | 0.0958 |
| Black | 1 | 0.0727 | 0.0649 | -0.0544 | 0.1998 | 1.26 | 0.2623 |
| Hispanic | 1 | 0.0621 | 0.0662 | -0.0677 | 0.1918 | 0.88 | 0.3484 |
| Asian | 1 | 0.0000 | 0.0000 | 0.0000 | 0.0000 | . | . |
| Other | 1 | 0.0316 | 0.0707 | -0.1069 | 0.1701 | 0.20 | 0.6549 |
| Bronx | 1 | 0.0630 | 0.0726 | -0.0794 | 0.2053 | 0.75 | 0.3858 |
| Brooklyn | 1 | -0.0282 | 0.0580 | -0.1419 | 0.0854 | 0.24 | 0.6263 |
| Manhattan | 0 | 0.0082 | 0.0993 | -0.1864 | 0.2028 | 0.01 | 0.9342 |
| Queens | 1 | 0.0000 | 0.0000 | 0.0000 | 0.0000 | . | . |
| Staten Island | 1 | -0.0228 | 0.0618 | -0.1439 | 0.0983 | 0.14 | 0.7121 |
| Male | 1 | 0.0320 | 0.0441 | -0.0544 | 0.1184 | 0.53 | 0.4680 |
| Female | 0 | 0.0000 | 0.0000 | 0.0000 | 0.0000 | . | . |
| US Born | 1 | -0.0502 | 0.0488 | -0.1459 | 0.0455 | 1.06 | 0.3036 |
| Non-US Born | 0 | 0.0000 | 0.0000 | 0.0000 | 0.0000 | . | . |

| **S1 Table: Raw Tables of the Six Poisson Regression Models (Mail Invitations Included)** | | | | | | | |
| --- | --- | --- | --- | --- | --- | --- | --- |
| **Demographic Characteristics** | **DF** | **Estimate** | **Standard Error** | **95% Lower Confidence Limit** | **95% Upper Confidence Limit** | **Wald Chi-Square** | **Pr > ChiSq** |
| **JUNE HOP** | | | | | | | |
| Intercept | 1 | 0.7637 | 0.1459 | 0.4777 | 1.0497 | 27.38 | <.0001 |
| 18-24 | 1 | -0.0989 | 0.1182 | -0.3305 | 0.1327 | 0.70 | 0.4026 |
| 25-44 | 0 | 0.0000 | 0.0000 | 0.0000 | 0.0000 | . | . |
| 45-64 | 1 | 0.0885 | 0.0558 | -0.0209 | 0.1979 | 2.51 | 0.1129 |
| 65+ | 1 | 0.3945 | 0.0584 | 0.2800 | 0.5091 | 45.58 | <.0001 |
| Less than high school | 0 | 0.0000 | 0.0000 | 0.0000 | 0.0000 | . | . |
| High school graduate | 1 | -0.0993 | 0.1349 | -0.3636 | 0.1650 | 0.54 | 0.4616 |
| Some college | 1 | -0.0792 | 0.1276 | -0.3294 | 0.1709 | 0.39 | 0.5348 |
| College graduate | 1 | -0.1038 | 0.1244 | -0.3476 | 0.1401 | 0.70 | 0.4042 |
| <200%FPL | 1 | -0.0195 | 0.0553 | -0.1278 | 0.0888 | 0.12 | 0.7238 |
| ≥200%FPL | 0 | 0.0000 | 0.0000 | 0.0000 | 0.0000 | . | . |
| White | 0 | 0.0000 | 0.0000 | 0.0000 | 0.0000 | . | . |
| Black | 1 | 0.0322 | 0.0759 | -0.1165 | 0.1809 | 0.18 | 0.6715 |
| Hispanic | 1 | 0.0336 | 0.0707 | -0.1050 | 0.1722 | 0.23 | 0.6349 |
| Asian | 1 | -0.0036 | 0.0718 | -0.1443 | 0.1372 | 0.00 | 0.9601 |
| Other | 1 | 0.1217 | 0.1259 | -0.1250 | 0.3685 | 0.93 | 0.3337 |
| Bronx | 1 | -0.0361 | 0.0824 | -0.1975 | 0.1253 | 0.19 | 0.6614 |
| Brooklyn | 1 | -0.0085 | 0.0598 | -0.1257 | 0.1088 | 0.02 | 0.8876 |
| Manhattan | 0 | 0.0000 | 0.0000 | 0.0000 | 0.0000 | . | . |
| Queens | 1 | -0.0191 | 0.0632 | -0.1430 | 0.1048 | 0.09 | 0.7627 |
| Staten Island | 1 | 0.0908 | 0.1066 | -0.1181 | 0.2998 | 0.73 | 0.3943 |
| Male | 1 | 0.0072 | 0.0470 | -0.0848 | 0.0993 | 0.02 | 0.8774 |
| Female | 0 | 0.0000 | 0.0000 | 0.0000 | 0.0000 | . | . |
| US Born | 1 | -0.0294 | 0.0526 | -0.1325 | 0.0737 | 0.31 | 0.5763 |
| Non-US Born | 0 | 0.0000 | 0.0000 | 0.0000 | 0.0000 | . | . |
