# Supplementary material for: Assessing targeted invitation and response modes to improve survey participation in a diverse New York City panel: Healthy NYC
Source: PLoS One. 2023 Jan 26;18(1):e0280911. doi: 10.1371/journal.pone.0280911 (PMC9879422; doi:10.1371/journal.pone.0280911)
Supplement: S2 Table — (DOCX) [file pone.0280911.s002.docx]

# **Supporting Information**

| **S2 Table: Raw Tables of the Six Poisson Regression Models (Email and Text Invitations Only)** | | | | | | | |
| --- | --- | --- | --- | --- | --- | --- | --- |
| **Demographic Characteristics** | **DF** | **Estimate** | **Standard Error** | **95% Lower Confidence Limit** | **95% Upper Confidence Limit** | **Wald Chi-Square** | **Pr > ChiSq** |
| **NOVEMBER SDH** | | | | | | | |
| Intercept | 1 | 0.8673 | 0.1108 | 0.6501 | 1.0845 | 61.26 | <.0001 |
| 18-24 | 1 | -0.0689 | 0.0770 | -0.2198 | 0.0821 | 0.80 | 0.3713 |
| 25-44 | 0 | 0.0000 | 0.0000 | 0.0000 | 0.0000 | . | . |
| 45-64 | 1 | -0.0563 | 0.0399 | -0.1344 | 0.0218 | 2.00 | 0.1576 |
| 65+ | 1 | -0.6427 | 0.1957 | -1.0264 | -0.2591 | 10.78 | 0.0010 |
| Less than high school | 0 | 0.0000 | 0.0000 | 0.0000 | 0.0000 | . | . |
| High school graduate | 1 | 0.0724 | 0.1058 | -0.1348 | 0.2797 | 0.47 | 0.4933 |
| Some college | 1 | 0.0344 | 0.1011 | -0.1637 | 0.2325 | 0.12 | 0.7337 |
| College graduate | 1 | 0.0044 | 0.1000 | -0.1917 | 0.2005 | 0.00 | 0.9651 |
| <200%FPL | 1 | -0.0916 | 0.0463 | -0.1823 | -0.0010 | 3.93 | 0.0475 |
| ≥200%FPL | 0 | 0.0000 | 0.0000 | 0.0000 | 0.0000 | . | . |
| White | 0 | 0.0000 | 0.0000 | 0.0000 | 0.0000 | . | . |
| Black | 1 | 0.1651 | 0.0597 | 0.0481 | 0.2821 | 7.65 | 0.0057 |
| Hispanic | 1 | 0.1253 | 0.0507 | 0.0260 | 0.2246 | 6.12 | 0.0134 |
| Asian | 1 | -0.0302 | 0.0529 | -0.1340 | 0.0735 | 0.33 | 0.5677 |
| Other | 1 | -0.0086 | 0.1049 | -0.2141 | 0.1969 | 0.01 | 0.9345 |
| Bronx | 1 | -0.0489 | 0.0630 | -0.1723 | 0.0745 | 0.60 | 0.4376 |
| Brooklyn | 1 | -0.0446 | 0.0438 | -0.1304 | 0.0411 | 1.04 | 0.3076 |
| Manhattan | 0 | 0.0000 | 0.0000 | 0.0000 | 0.0000 | . | . |
| Queens | 1 | 0.0268 | 0.0489 | -0.0691 | 0.1227 | 0.30 | 0.5838 |
| Staten Island | 1 | 0.0923 | 0.0796 | -0.0636 | 0.2482 | 1.35 | 0.2458 |
| Male | 1 | 0.0676 | 0.0369 | -0.0046 | 0.1399 | 3.37 | 0.0665 |
| Female | 0 | 0.0000 | 0.0000 | 0.0000 | 0.0000 | . | . |
| US Born | 1 | -0.0623 | 0.0394 | -0.1395 | 0.0149 | 2.51 | 0.1135 |
| Non-US Born | 0 | 0.0000 | 0.0000 | 0.0000 | 0.0000 | . | . |

| **S2 Table: Raw Tables of the Six Poisson Regression Models (Email and Text Invitations Only)** | | | | | | | |
| --- | --- | --- | --- | --- | --- | --- | --- |
| **Demographic Characteristics** | **DF** | **Estimate** | **Standard Error** | **95% Lower Confidence Limit** | **95% Upper Confidence Limit** | **Wald Chi-Square** | **Pr > ChiSq** |
| **DECEMBER COVID** | | | | | | | |
| Intercept | 1 | 1.0214 | 0.1843 | 0.6601 | 1.3827 | 30.71 | <.0001 |
| 18-24 | 1 | -0.0502 | 0.1016 | -0.2493 | 0.1489 | 0.24 | 0.6210 |
| 25-44 | 0 | 0.0000 | 0.0000 | 0.0000 | 0.0000 | . | . |
| 45-64 | 1 | 0.1105 | 0.0642 | -0.0154 | 0.2363 | 2.96 | 0.0853 |
| 65+^**^ | 1 | -- | -- | -- | -- | -- | -- |
| Less than high school | 0 | 0.0000 | 0.0000 | 0.0000 | 0.0000 | . | . |
| High school graduate | 1 | -0.1573 | 0.1682 | -0.4869 | 0.1723 | 0.87 | 0.3496 |
| Some college | 1 | -0.2423 | 0.1648 | -0.5653 | 0.0807 | 2.16 | 0.1416 |
| College graduate | 1 | -0.2760 | 0.1597 | -0.5890 | 0.0370 | 2.99 | 0.0840 |
| <200%FPL | 1 | -0.0639 | 0.0698 | -0.2007 | 0.0729 | 0.84 | 0.3598 |
| ≥200%FPL | 0 | 0.0000 | 0.0000 | 0.0000 | 0.0000 | . | . |
| White | 0 | 0.0000 | 0.0000 | 0.0000 | 0.0000 | . | . |
| Black | 1 | 0.1028 | 0.0915 | -0.0765 | 0.2822 | 1.26 | 0.2612 |
| Hispanic | 1 | -0.0699 | 0.0808 | -0.2283 | 0.0885 | 0.75 | 0.3873 |
| Asian | 1 | -0.0090 | 0.0781 | -0.1621 | 0.1441 | 0.01 | 0.9081 |
| Other | 1 | -0.1587 | 0.1780 | -0.5076 | 0.1902 | 0.79 | 0.3726 |
| Bronx | 1 | 0.1814 | 0.0904 | 0.0043 | 0.3585 | 4.03 | 0.0447 |
| Brooklyn | 1 | -0.0240 | 0.0721 | -0.1653 | 0.1173 | 0.11 | 0.7389 |
| Manhattan | 0 | 0.0000 | 0.0000 | 0.0000 | 0.0000 | . | . |
| Queens | 1 | 0.0702 | 0.0804 | -0.0874 | 0.2277 | 0.76 | 0.3828 |
| Staten Island | 1 | 0.1416 | 0.1539 | -0.1599 | 0.4432 | 0.85 | 0.3574 |
| Male | 1 | -0.0257 | 0.0580 | -0.1394 | 0.0880 | 0.20 | 0.6579 |
| Female | 0 | 0.0000 | 0.0000 | 0.0000 | 0.0000 | . | . |
| US Born | 1 | -0.0093 | 0.0632 | -0.1333 | 0.1146 | 0.02 | 0.8825 |
| Non-US Born | 0 | 0.0000 | 0.0000 | 0.0000 | 0.0000 | . | . |

^** For the December COVID survey, there too few panelists in the 65+ age category who received exclusively email or text invites and, therefore, there were insufficient data points to perform the analysis.^

| **S2 Table: Raw Tables of the Six Poisson Regression Models (Email and Text Invitations Only)** | | | | | | | |
| --- | --- | --- | --- | --- | --- | --- | --- |
| **Demographic Characteristics** | **DF** | **Estimate** | **Standard Error** | **95% Lower Confidence Limit** | **95% Upper Confidence Limit** | **Wald Chi-Square** | **Pr > ChiSq** |
| **JANUARY MH** | | | | | | | |
| Intercept | 1 | 0.8444 | 0.1517 | 0.5470 | 1.1418 | 30.97 | <.0001 |
| 18-24 | 1 | -0.2439 | 0.1149 | -0.4692 | -0.0186 | 4.50 | 0.0338 |
| 25-44 | 0 | 0.0000 | 0.0000 | 0.0000 | 0.0000 | . | . |
| 45-64 | 1 | 0.0773 | 0.0575 | -0.0353 | 0.1900 | 1.81 | 0.1785 |
| 65+ | 1 | -0.2332 | 0.2475 | -0.7183 | 0.2519 | 0.89 | 0.3460 |
| Less than high school | 0 | 0.0000 | 0.0000 | 0.0000 | 0.0000 | . | . |
| High school graduate | 1 | 0.0371 | 0.1487 | -0.2543 | 0.3284 | 0.06 | 0.8031 |
| Some college | 1 | 0.0788 | 0.1415 | -0.1986 | 0.3561 | 0.31 | 0.5778 |
| College graduate | 1 | 0.1295 | 0.1392 | -0.1433 | 0.4022 | 0.87 | 0.3521 |
| <200%FPL | 1 | 0.1043 | 0.0645 | -0.0220 | 0.2307 | 2.62 | 0.1056 |
| ≥200%FPL | 0 | 0.0000 | 0.0000 | 0.0000 | 0.0000 | . | . |
| White | 0 | 0.0000 | 0.0000 | 0.0000 | 0.0000 | . | . |
| Black | 1 | 0.1520 | 0.0820 | -0.0087 | 0.3127 | 3.44 | 0.0638 |
| Hispanic | 1 | -0.0080 | 0.0727 | -0.1506 | 0.1346 | 0.01 | 0.9128 |
| Asian | 1 | 0.0916 | 0.0756 | -0.0566 | 0.2399 | 1.47 | 0.2258 |
| Other | 1 | -0.1632 | 0.1484 | -0.4542 | 0.1277 | 1.21 | 0.2715 |
| Bronx | 1 | 0.0654 | 0.0837 | -0.0987 | 0.2296 | 0.61 | 0.4347 |
| Manhattan | 0 | 0.0000 | 0.0000 | 0.0000 | 0.0000 | . | . |
| Brooklyn | 1 | -0.0369 | 0.0658 | -0.1658 | 0.0919 | 0.32 | 0.5742 |
| Queens | 1 | -0.0405 | 0.0710 | -0.1796 | 0.0986 | 0.33 | 0.5681 |
| Staten Island | 1 | -0.0916 | 0.1407 | -0.3673 | 0.1841 | 0.42 | 0.5150 |
| Male | 1 | 0.0217 | 0.0523 | -0.0808 | 0.1242 | 0.17 | 0.6780 |
| Female | 0 | 0.0000 | 0.0000 | 0.0000 | 0.0000 | . | . |
| US Born | 1 | -0.0172 | 0.0579 | -0.1308 | 0.0964 | 0.09 | 0.7668 |
| Non-US Born | 0 | 0.0000 | 0.0000 | 0.0000 | 0.0000 | . | . |

| **S2 Table: Raw Tables of the Six Poisson Regression Models (Email and Text Invitations Only)** | | | | | | | |
| --- | --- | --- | --- | --- | --- | --- | --- |
| **Demographic Characteristics** | **DF** | **Estimate** | **Standard Error** | **95% Lower Confidence Limit** | **95% Upper Confidence Limit** | **Wald Chi-Square** | **Pr > ChiSq** |
| **FEBRUARY COVID** | | | | | | | |
| Intercept | 1 | 1.0581 | 0.1707 | 0.7235 | 1.3927 | 38.42 | <.0001 |
| 18-24 | 1 | -0.1553 | 0.1335 | -0.4169 | 0.1063 | 1.35 | 0.2446 |
| 25-44 | 0 | 0.0000 | 0.0000 | 0.0000 | 0.0000 | . | . |
| 45-64 | 1 | 0.0113 | 0.0579 | -0.1021 | 0.1247 | 0.04 | 0.8453 |
| 65+ | 1 | 0.1956 | 0.4195 | -0.6265 | 1.0178 | 0.22 | 0.6409 |
| Less than high school | 0 | 0.0000 | 0.0000 | 0.0000 | 0.0000 | . | . |
| High school graduate | 1 | -0.1461 | 0.1656 | -0.4707 | 0.1785 | 0.78 | 0.3777 |
| Some college | 1 | -0.0409 | 0.1529 | -0.3406 | 0.2588 | 0.07 | 0.7892 |
| College graduate | 1 | -0.1352 | 0.1510 | -0.4311 | 0.1606 | 0.80 | 0.3703 |
| <200%FPL | 1 | -0.1558 | 0.0700 | -0.2929 | -0.0187 | 4.96 | 0.0260 |
| ≥200%FPL | 0 | 0.0000 | 0.0000 | 0.0000 | 0.0000 | . | . |
| White | 0 | 0.0000 | 0.0000 | 0.0000 | 0.0000 | . | . |
| Black | 1 | 0.0491 | 0.0937 | -0.1346 | 0.2328 | 0.27 | 0.6003 |
| Hispanic | 1 | 0.1055 | 0.0854 | -0.0619 | 0.2728 | 1.53 | 0.2167 |
| Asian | 1 | 0.0504 | 0.0807 | -0.1078 | 0.2087 | 0.39 | 0.5321 |
| Other | 1 | -0.2299 | 0.1837 | -0.5900 | 0.1302 | 1.57 | 0.2109 |
| Bronx | 1 | 0.0120 | 0.0981 | -0.1803 | 0.2043 | 0.01 | 0.9029 |
| Brooklyn | 1 | -0.0669 | 0.0726 | -0.2091 | 0.0753 | 0.85 | 0.3565 |
| Manhattan | 0 | 0.0000 | 0.0000 | 0.0000 | 0.0000 | . | . |
| Queens | 1 | -0.0531 | 0.0812 | -0.2123 | 0.1062 | 0.43 | 0.5137 |
| Staten Island | 1 | 0.0699 | 0.1254 | -0.1759 | 0.3157 | 0.31 | 0.5772 |
| Male | 1 | 0.0539 | 0.0572 | -0.0582 | 0.1660 | 0.89 | 0.3461 |
| Female | 0 | 0.0000 | 0.0000 | 0.0000 | 0.0000 | . | . |
| US Born | 1 | -0.1062 | 0.0621 | -0.2279 | 0.0155 | 2.92 | 0.0872 |
| Non-US Born | 0 | 0.0000 | 0.0000 | 0.0000 | 0.0000 | . | . |

| **S2 Table: Raw Tables of the Six Poisson Regression Models (Email and Text Invitations Only)** | | | | | | | |
| --- | --- | --- | --- | --- | --- | --- | --- |
| **Demographic Characteristics** | **DF** | **Estimate** | **Standard Error** | **95% Lower Confidence Limit** | **95% Upper Confidence Limit** | **Wald Chi-Square** | **Pr > ChiSq** |
| **MARCH HOP** | | | | | | | |
| Intercept | 1 | 0.4530 | 0.1646 | 0.1304 | 0.7757 | 7.57 | 0.0059 |
| 18-24 | 1 | -0.0014 | 0.1175 | -0.2317 | 0.2289 | 0.00 | 0.9904 |
| 25-44 | 0 | 0.0000 | 0.0000 | 0.0000 | 0.0000 | . | . |
| 45-64 | 1 | 0.0454 | 0.0638 | -0.0797 | 0.1706 | 0.51 | 0.4765 |
| 65+ | 1 | 0.1442 | 0.1535 | -0.1566 | 0.4450 | 0.88 | 0.3476 |
| Less than high school | 0 | 0.0000 | 0.0000 | 0.0000 | 0.0000 | . | . |
| High school graduate | 1 | -0.0537 | 0.1589 | -0.3651 | 0.2577 | 0.11 | 0.7354 |
| Some college | 1 | -0.0216 | 0.1475 | -0.3106 | 0.2674 | 0.02 | 0.8836 |
| College graduate | 1 | -0.0316 | 0.1456 | -0.3169 | 0.2537 | 0.05 | 0.8280 |
| <200%FPL | 1 | -0.0613 | 0.0715 | -0.2016 | 0.0789 | 0.73 | 0.3913 |
| ≥200%FPL | 0 | 0.0000 | 0.0000 | 0.0000 | 0.0000 | . | . |
| White | 0 | 0.0000 | 0.0000 | 0.0000 | 0.0000 | . | . |
| Black | 1 | 0.1611 | 0.1028 | -0.0403 | 0.3625 | 2.46 | 0.1169 |
| Hispanic | 1 | 0.1488 | 0.0828 | -0.0135 | 0.3112 | 3.23 | 0.0723 |
| Asian | 1 | 0.0288 | 0.0867 | -0.1412 | 0.1987 | 0.11 | 0.7400 |
| Other | 1 | 0.0067 | 0.1605 | -0.3080 | 0.3213 | 0.00 | 0.9668 |
| Bronx | 1 | -0.0873 | 0.1022 | -0.2875 | 0.1130 | 0.73 | 0.3930 |
| Brooklyn | 1 | -0.0117 | 0.0730 | -0.1548 | 0.1314 | 0.03 | 0.8724 |
| Manhattan | 0 | 0.0000 | 0.0000 | 0.0000 | 0.0000 | . | . |
| Queens | 1 | 0.0296 | 0.0782 | -0.1236 | 0.1829 | 0.14 | 0.7045 |
| Staten Island | 1 | 0.0125 | 0.1585 | -0.2981 | 0.3232 | 0.01 | 0.9371 |
| Male | 1 | -0.0254 | 0.0602 | -0.1433 | 0.0925 | 0.18 | 0.6729 |
| Female | 0 | 0.0000 | 0.0000 | 0.0000 | 0.0000 | . | . |
| US Born | 1 | 0.0242 | 0.0675 | -0.1081 | 0.1564 | 0.13 | 0.7202 |
| Non-US Born | 0 | 0.0000 | 0.0000 | 0.0000 | 0.0000 | . | . |

| **S2 Table: Raw Tables of the Six Poisson Regression Models (Email and Text Invitations Only)** | | | | | | | |
| --- | --- | --- | --- | --- | --- | --- | --- |
| **Demographic Characteristics** | **DF** | **Estimate** | **Standard Error** | **95% Lower Confidence Limit** | **95% Upper Confidence Limit** | **Wald Chi-Square** | **Pr > ChiSq** |
| **JUNE HOP** | | | | | | | |
| Intercept | 1 | 0.8762 | 0.1744 | 0.5344 | 1.2180 | 25.25 | <.0001 |
| 18-24 | 1 | -0.0968 | 0.1189 | -0.3299 | 0.1362 | 0.66 | 0.4155 |
| 25-44 | 0 | 0.0000 | 0.0000 | 0.0000 | 0.0000 | . | . |
| 45-64 | 1 | 0.0194 | 0.0613 | -0.1007 | 0.1394 | 0.10 | 0.7517 |
| 65+ | 1 | 0.2398 | 0.4538 | -0.6495 | 1.1292 | 0.28 | 0.5971 |
| Less than high school | 0 | 0.0000 | 0.0000 | 0.0000 | 0.0000 | . | . |
| High school graduate | 1 | -0.1587 | 0.1639 | -0.4800 | 0.1626 | 0.94 | 0.3331 |
| Some college | 1 | -0.1621 | 0.1578 | -0.4715 | 0.1473 | 1.05 | 0.3045 |
| College graduate | 1 | -0.1988 | 0.1537 | -0.5000 | 0.1024 | 1.67 | 0.1958 |
| <200%FPL | 1 | -0.0066 | 0.0690 | -0.1419 | 0.1287 | 0.01 | 0.9239 |
| ≥200%FPL | 0 | 0.0000 | 0.0000 | 0.0000 | 0.0000 | . | . |
| White | 0 | 0.0000 | 0.0000 | 0.0000 | 0.0000 | . | . |
| Black | 1 | 0.0166 | 0.0943 | -0.1682 | 0.2014 | 0.03 | 0.8601 |
| Hispanic | 1 | 0.0299 | 0.0802 | -0.1274 | 0.1872 | 0.14 | 0.7092 |
| Asian | 1 | -0.0058 | 0.0796 | -0.1619 | 0.1503 | 0.01 | 0.9418 |
| Other | 1 | 0.1275 | 0.1427 | -0.1521 | 0.4070 | 0.80 | 0.3716 |
| Bronx | 1 | -0.0652 | 0.0998 | -0.2608 | 0.1305 | 0.43 | 0.5138 |
| Brooklyn | 1 | -0.0397 | 0.0727 | -0.1823 | 0.1028 | 0.30 | 0.5847 |
| Manhattan | 0 | 0.0000 | 0.0000 | 0.0000 | 0.0000 | . | . |
| Queens | 1 | -0.0413 | 0.0790 | -0.1962 | 0.1135 | 0.27 | 0.6007 |
| Staten Island | 1 | 0.0679 | 0.1450 | -0.2163 | 0.3521 | 0.22 | 0.6396 |
| Male | 1 | 0.0246 | 0.0592 | -0.0914 | 0.1407 | 0.17 | 0.6775 |
| Female | 0 | 0.0000 | 0.0000 | 0.0000 | 0.0000 | . | . |
| US Born | 1 | -0.0487 | 0.0604 | -0.1670 | 0.0697 | 0.65 | 0.4203 |
| Non-US Born | 0 | 0.0000 | 0.0000 | 0.0000 | 0.0000 | . | . |
